# Hepatitis B vaccine uptake among healthcare workers in a referral hospital, Accra

**DOI:** 10.11604/pamj.2019.33.96.18042

**Published:** 2019-06-10

**Authors:** Gloria Akosua Ansa, Kenneth Nana Affoh Ofori, Ekua Essumanma Houphouet, Afua Asabea Amoabeng, Jerry Selase Sifa, Christian Kofi Amenuveve, Gifty Harriet Odame

**Affiliations:** 1 University of Ghana Health Services, Legon, Accra, Ghana; 2 Columbia University College of Physicians and Surgeon, New York, USA

**Keywords:** Hepatitis B, vaccination, uptake, healthcare workers, University, sub-Saharan Africa, Ghana

## Abstract

**Introduction:**

Hepatitis B vaccination among healthcare workers (HCWs) in Ghana has not been actively pursued despite the endemicity of the infection. This study measures the hepatitis B vaccine uptake among HCWs at the University of Ghana Hospital, Legon (UGHL) and identifies the factors associated with vaccination.

**Methods:**

An analytical cross-sectional study involving all staff who have direct contact with patients was conducted. Self-administered questionnaires were used to collect data on vaccination status, age, sex, type of staff, duration of work in the facility, exposure to blood or blood products, blood stained linens/waste, sharp instruments and performance of invasive procedures. Data was analysed using STATA 14. Continuous variables were described using median values and interquartile ranges (IQR) and categorical variables as proportions. Bivariate and multivariate analysis were conducted to identify the factors associated with hepatitis B vaccination status.

**Results:**

Of the 161 participants interviewed, 63.4% were females with median age 35 years (IQR: 27-45). Eighty-six (53.4%) of the respondents had taken the hepatitis B vaccine with 79.1% of them having completed the vaccination schedule. Factors associated with vaccination were working for more than 16 years (OR: 3.8, CI: 1.02-12.72), daily exposure to blood/blood products (OR: 4.1, CI: 1.43-11.81) and sharp instruments (OR: 4.45, CI: 1.39- 14.24), performing invasive procedures daily (OR: 3.0, CI: 1.07-8.45) and frequent exposure to blood stained linens/waste (OR: 6.1, CI: 1.41-26.51).

**Conclusion:**

The lack of hepatitis B vaccination among some HCWs at UGHL puts them at risk of contracting hepatitis B infection.

## Introduction

Hepatitis B virus (HBV) infection affects the liver and can result in acute or chronic liver disease [1]. The disease is a major public health concern currently affecting about 2 billion people globally and resulting in chronic infection in over 320 million people [2]. The infection and its complications continue to be a major cause of mortality [3,4]. Hepatitis B prevalence is highest in sub-Saharan Africa and East Asia, where between 5-10% of the adult population is chronically infected [1,5] and the lifetime risk of acquiring the infection is greater than 60% [2]. HBV infection has long been recognised as an occupational risk for health care personnel [6-8]. It is estimated that 5.9% of health care workers (HCWs) worldwide are exposed to hepatitis B infection annually [9] and the risk of infection after exposure to highly infectious fluids such as blood from a patient who is actively transmitting the disease is up to 30% [10-12]. Although the infection is vaccine preventable, studies in parts of Africa have shown low uptake of the hepatitis B vaccine among HCWs [13-15]. In sub-Saharan Africa, majority of studies report 35% to 65% of HCWs have ever received the vaccine and an even lower proportion have completed the vaccination schedule. The vaccination coverage was however approximately 92% in a hospital in Nigeria which had introduced a hepatitis B campaign for all its HCWs [16]. Hepatitis B infection is endemic in Ghana with sero-prevalence in different parts of the country ranging from 6.7% to 14.7% in blood donors [17-20], 10.6% to 14.3% in pregnant women [17,21-23] and 15.6% in children [24]. Despite the paucity of data on prevalence of the infection among HCWs in the country, few studies in different parts of the country have looked at the knowledge and attitude of HCW towards hepatitis B and their uptake of vaccination against the infection [25,26]. A study conducted in the Tamale Metropolis found 44.4% of nurses in the metropolis had taken at least a dose of the hepatitis B vaccine with a quarter of them not completing the vaccination schedule [25]. Another study assessing the knowledge, attitude and practices of staff on hepatitis B in a district hospital in the Ashanti Region of Ghana found about 70% of staff had taken the vaccine [26]. Hepatitis B infection prevention in the general population has increasingly been of national interest over the past 2 decades. Consequently, the pentavalent vaccine which includes the hepatitis B virus was introduced in Ghana's Expanded Programme on Immunization (EPI) for children in the year 2002 [27,28]. In Ghana the recommended adult vaccination protocol is 0.5ml of the vaccine given in 3 doses at months 0, 1 and 6. Hepatitis B vaccines are readily available in health facilities across Accra, the nation's capital, and the University of Ghana Hospital Legon (UGHL) has had this service available since the year 2016. However, prevention of HBV infection among HCWs has not been actively pursued in the country, just like in many other resource poor settings. This is evidenced by the lack of a structured vaccination plan for HCWs and the few studies on vaccine uptake among HCWs in Ghana. We therefore evaluated the hepatitis B vaccine coverage and the factors associated with vaccination status among HCWs at University of Ghana Hospital, Legon as a basis for advocacy and development of a more comprehensive vaccination plan for health workers in the facility.

## Methods

**Study design**: an analytical cross-sectional study was conducted at the UGHL. Data was collected from HCWs at the facility using a structured questionnaire from July to December 2016.

**Study setting**: the UGHL is a 140-bed hospital, situated at Legon in Accra, Ghana. It is a quasi-government hospital which operates as one of the departments in the University of Ghana, Legon. Originally established to cater for the health needs of the student population, staff and their dependents, it has now assumed the function of a district hospital and a referral centre serving the Legon environs and beyond. The facility attends to a student population of about 38,000, approximately 5,774 staff, 6,107 registered dependents and other inhabitants in its catchment area. At the end of the year 2017 the hospital attended to approximately 8,334 patients at its out-patient department and admitted about 500 monthly. Services provided by the hospital include general practice and care in over 13 specialties including internal medicine, emergency medicine, general surgery, obstetrics and gynaecology, paediatrics, public health and physiotherapy. The hospital has a total of 302 permanent staff. Of these, 232 are likely to have direct contact with patients and/or their body fluids as a result of their work. These include doctors, physician assistants, nurses, laboratory staff, cleaners, morgue attendants and laundry staff. Testing for hepatitis B is free for staff under the university's free medical cover for its employees. Hepatitis B vaccination is however offered at a cost at its Public Health Unit. In 2004 however, a hospital wide hepatitis B vaccination was conducted at no cost to staff.

**Study population**: the study population included all staff of the UGHL.

**Inclusion criteria**: staff of the UGHL who have direct contact with patients or their body fluids. Direct contact was defined as touching the patient, their body fluids or wastes, instruments used on the patients or the linens used by patients.

**Exclusion criteria**: staff with a history of hepatitis B infection.

**Study variables**: the dependent variable was hepatitis B vaccination status and was measured as “yes” if the participant had taken at least one dose of the hepatitis B vaccine and “No” if they had not taken any dose. The independent variables measured were age, sex, staff type (whether clinical or non-clinical), duration of work in the hospital and in current capacity, frequency of exposure to blood or blood products, blood-stained linen, sharp instruments and frequency of performance of surgical or invasive procedure. Frequency of exposure to these risks was measured by asking participants to indicate whether they were never exposed or exposed on average about once a week, more than once a week or every day. These were further categorized as never exposed if they were never exposed to the risk, occasionally exposed if they were exposed on average once a week, frequently exposed if they were exposed more than once a week and exposed daily if they were exposed every day.

**Data collection**: self-administered questionnaires were given to all eligible participants in their respective departments. Two hundred and four (204) questionnaires were given out as the remaining staff were either unavailable during the period of the study or were unwilling to participate. Research assistants then did a follow up visit to the participants to pick up filled questionnaire. Out of the 204 surveys delivered, one hundred and sixty-one (161) completed surveys were returned giving a response rate of 78.9% in this HCW population.

**Data analysis**: data was entered into Microsoft Excel for cleaning and then transferred into STATA14 statistical software for analysis. Socio-demographic characteristics of participants were summarized as frequencies and proportions. Continuous variables such as age and duration of work in the hospital and in current capacity were summarized using median and interquartile ranges (IQR). Bivariate analysis was done to identify factors that are associated with taking the vaccine. The significant factors were included in a multiple logistic regression model to identify the factors predicting vaccination status. Findings from the regression were reported as unadjusted and adjusted odds ratios. The analysis was done at 95% confidence level and p values were considered significant if p<0.05.

**Ethical considerations**: ethical approval for the study was obtained from the Ethical Review Board of Noguchi Memorial Institute for Medical Research (reference number 101/15-16). Permission was also sought from the Director of the University of Ghana Hospital Legon and the various heads of departments at UGHL prior to the start of the study. Written informed consent was sought from participants prior to their inclusion in the study.

## Results

**Participant characteristics**: majority 102 (63.4%) of the participants were female with approximately two-thirds 96 (64%) aged between 20 and 34 years. Forty-one percent (41%) were non-clinical staff. The median age for the participants was 31years (IQR: 27-45). The median duration participants had worked at the facility and that spent in current capacity were 5 years (IQR: 1-10) and 4 years (IQR: 0.8-8) respectively. About a third 53 (34.9%) had worked for less than 1 year and a fifth 28 (18.4%) for more than 10 years. Approximately a quarter of the respondents 34 (23.9%) had worked in their current capacities for 1 to 5 years. Those who had worked for 16 years or more were 14.1% (Table 1).

**Table 1:** Demographic characteristics of participants

Variable	Frequency	Proportion (%)
**Age (Years)**	N=150	
20-34	96	64.0
35-49	29	19.3
50-64	25	16.7
**Sex**	N=161	
Female	102	63.4
Male	59	36.6
**Staff Type**	N=156	
Clinical	92	59.0
Non-clinical	64	41.0
**Length of time working in the hospital (years)**	N=152	
Less than1	53	34.9
1-5	35	23.0
6-10	36	23.7
11-15	10	6.6
16 or more	18	11.8
**Length of time working in current capacity (years)**	N=142	
Less than1	16	11.2
1-5	34	23.9
6-10	32	22.5
11-15	10	7.0
16 or more	20	14.1

**Hepatitis B vaccine coverage**: nearly half of the participants 75 (46.6%) had not received the hepatitis B vaccine. Among the 86 who had received the vaccine, 79.1% had received all three doses (Table 2).

**Table 2:** Hepatitis B vaccine coverage and the number of vaccine doses received among HCWs at UGHL

Hepatitis B vaccination status	Frequency(N)	Proportion (%)
Taken Hepatitis B vaccine	86	53.4
Not taken Hepatitis B vaccine	75	46.6
**Total**	**161**	**100**
**Vaccination status**		
One HB vaccine dose	5	5.8
Two HB vaccine doses	13	15.1
Full vaccination (3 doses)	68	79.1
**Total**	**86**	**100%**

**Factors associated with vaccination status**: the odds of having worked for 16 years or more among those who had been vaccinated against hepatitis B was 3.8 times higher compared to those who had not vaccinated (CI: 1.02-12.72). Those who were exposed to blood and blood products daily were 4.1 times as likely to have received the vaccine compared to those who were never exposed (CI:1.43-11.81). The odds of being exposed to sharp instruments daily among those vaccinated was 4.45 times higher than in those who were not vaccinated. Staff who performed surgical/invasive procedures frequently were 9.7 times (CI: 2.69-35.09) more likely to have taken the hepatitis B vaccine compared to those who never performed these procedures. Those who performed the surgical/invasive procedures daily were also 3 times more likely (CI: 1.07-8.45) to have taken the hepatitis B vaccine. Compared to those who were never exposed to blood-stained linens and waste, those who were exposed frequently were 6.1 times more likely to have taken the vaccine. Frequently being exposed to blood and blood products was not significantly associated with vaccinating against hepatitis B after adjusting for duration of work, exposure to sharp instruments, performance of surgical or invasive procedures and exposure to blood stained linens and waste (Table 3).

**Table 3:** Factors associated with vaccination status among HCWs at UGHL

Variable	OR	95%CI	aOR	95% CI
**Length of time working in the hospital (years)**				
Less than 1	Ref		Ref	
1-5	2.3	0.95-5.60	2.41	0.97-6.03
6-10	1.2	0.51-2.82	1.23	0.51-2.97
11-15	0.80	0.20-3.18	0.73	0.17-3.09
16 or more	4.2*	1.22-14.55	3.82*	1.08-13.50
**Exposure to blood /blood product**				
Never	Ref		Ref	
Occasionally	1.98	0.68-5.79	1.95	0.59-6.46
Frequently	3.86*	1.15-12.91	3.25	0.83-12.71
Daily	4.75*	1.76-12.82	4.11*	1.43-11.81
**Exposure to sharp objects**				
Never	Ref		Ref	
Occasionally	2.37	0.75-7.52	1.65	0.46-5.90
Frequently	5.04*	1.46-17.37	2.90	0.71-11.90
Daily	5.48*	1.83-16.37	4.45*	1.39-14.24
**Involvement in surgical/invasive Procedures**				
Never	Ref		Ref	
Occasionally	2.03	0.82-4.99	2.12	0.82-5.48
Frequently	9.64*	2.75-33.75	9.71*	2.69-35.09
Daily	3.08*	1.11-8.55	3.01*	1.07-8.45
**Exposure to blood-stained linens/waste**				
Never	Ref		Ref	
Occasionally	1.85	0.59-4.16	1.85	0.59-5.75
Frequently	6.3*	1.63-24.39	6.11*	1.41-26.51
Daily	2.84*	1.08-745	2.66	0.95-7.43

OR-Odds ratio, aOR- adjusted odds ratio, CI - confidence interval

* Significant Odds ratios

## Discussion

We found that the uptake of hepatitis B vaccination among healthcare workers at University of Ghana Hospital Legon was low (53%) compared to WHO recommendation of vaccination for all high risk groups including HCWs with direct contact with patients or their body fluids [29]. This means that about half of the HCWs who have direct contact with patients and/or their body fluids do not have immunity against hepatitis B and can potentially be infected if they are exposed to patients who are actively transmitting hepatitis B infection. The low coverage observed could be due to the lack of guidelines on checking of hepatitis B status and vaccination for HCWs prior to being employed in the facility. Knowing one's hepatitis B status and vaccination against the infection are not prerequisite for employment in public health facilities in Ghana, including UGHL. HCWs may therefore vaccinate when they perceive themselves to be at risk of getting infected and when they can and want to commit resources to the process. This is contrary to the World Health Organization's recommendation that countries develop guidelines on hepatitis B vaccination for high risk groups such as HCWs who have direct contact with patients [29].

The hepatitis B vaccination coverage seen at UGHL is comparable to that in some parts of Africa [30] but also higher than in other countries within the region [31,32] where vaccination against hepatitis B infection is left largely to the discretion of the HCW. For instance, studies carried out among HCWs in a provincial hospital in Kenya and a University Hospital in Ethiopia reported lower coverages of 42% and 28% respectively [33,34], which are significantly lower than what was seen at UGHL, Ghana. Some studies have however found high coverages in some institutions in developing countries. In a University Teaching Hospital in Nigeria where the management had purchased the hepatitis B vaccine and offered them at no cost to the employees, the vaccine coverage recorded was over 90% among its HCW [16]. Ziglam *et al* (2013) have reported that coverage for the vaccine was 78% among HCWs in a tertiary facility in Libya. This coverage is therefore higher than what we observed among HCW in UGHL Ghana.

Whereas sub-Saharan Africa generally struggles with low hepatitis B vaccination coverage, studies among HCWs in developed countries tend to report coverages higher than 65% [35-38]. In countries like Croatia where hepatitis B vaccination is mandatory for all HCWs, coverage is as high as 98% [39]. Western countries have higher coverages because there are policies that make it mandatory for HCWs to vaccinate and obtain post vaccination follow up care [40-44]. Some of these countries have developed information targeted at HCW education and setting guidelines to allow the HCWs to follow their own care [45]. With about half of HCWs at UGHL not vaccinated against hepatitis B, and an even lower proportion (42%) having completed all three doses of the vaccination, the HCWs continue to be at risk of HBV infection because of their exposure to blood and body fluids. According to WHO, a safe working environment for health-care workers should include the offer of HBV immunization [3]. Healthcare facilities therefore have to work in conjunction with their supervising agencies to ensure HCWs are offered testing and vaccination in order to reduce the risk of nosocomial transmission of hepatitis B to them and ultimately reduce the transmission to other previously uninfected patients.

Health workers who had worked for 16 years or more in the hospital were 3.6 times more likely to be vaccinated than workers with less than a year's experience. This may be because these HCWs may have learned to appreciate their risk of being infected with hepatitis B better with longer years of service as they experience occupational accidents such as needle stick injuries during their work. Our finding may also be attributed to the fact that in addition to the appreciation of their risk, a hospital-wide vaccination for Hepatitis B was last conducted in 2004 giving the HCWs present at the time the opportunity to receive the vaccinations. This may suggest that when HCWs are actively offered the vaccination they may utilize it. This reasoning is affirmed by findings from a survey done in a national hospital in Tanzania which found that one of the reasons HCWs gave for not vaccinating was that the vaccination had not been offered to them [14]. Our finding of an association between duration of service and vaccination status also corroborates the findings of Abebaw *et al* (2017) who found that HCWs who had been working for 10 years or more were 12 times more likely to be vaccinated in a health facility based survey conducted in Ethiopia [46]. Staff at UGHL who were exposed to blood or blood products daily were more likely to vaccinate compared to those who were never exposed. Those who worked with sharp instruments daily were also more likely to vaccinate compared to those who never did. Those who performed surgical procedures daily or frequently were also more likely to be vaccinated when compared with those who never performed these procedures. These results were likely obtained because these categories of staff perceived themselves to be at a higher risk of getting infected and so took steps to get vaccinated. Perception of risk was identified to influence vaccine uptake in a study in two health facilities in Georgia [47]. This is also in line with findings from Tanzania where hepatitis B vaccination coverage was 57% even though vaccination was free for all health workers [14]. Among HCWs in Kenya, a study assessing the prevalence of percutaneous injury, cited low risk perception as a reason for not taking the vaccination [34]. This suggests that HCW sensitization on risk of getting hepatitis B infection may be necessary to improve vaccine uptake even when the vaccine is offered to them at no cost.

Daily exposure to blood stained linens was not significantly associated with vaccination status after adjusting for exposure to blood and blood products, sharp instruments and performance of surgical procedures. A key limitation of the study was that the current hepatitis B status was not assessed for participants prior to enrolment in the study and so it is possible a HCW with hepatitis B infection may have been enrolled. This is however unlikely as a hepatitis B screening exercise had been carried out by the UGHL for its staff in 2016, the same year as the study was conducted. Participants hepatitis B status is therefore unlikely to have changed significantly prior to their participation in the study. The study also relied on self-report of the participants' vaccination status as some participants had lost their immunization records. This is not expected to affect the findings of the study as participants were aware hepatitis B vaccination was not mandatory for their employment and hence declaring their status would not influence their employment in any way. In addition, there were no identifiers linking the responses to the participants and hence they were likely to have given their true vaccination status.

## Conclusion

This study found low hepatitis B vaccine coverage among HCW of UGHL, an indication that a large proportion of HCWs are at risk of getting infected with hepatitis B when they have contact with potentially infected patients. The factors associated with taking the vaccine included long duration of work in the facility, exposure to blood and blood products, exposure to sharp instruments and performance of surgical procedures. The low coverage observed may be due to several factors including the lack of clear policy on hepatitis B testing, vaccination and follow up care for HCWs prior to and during the period of their employment. We recommend the management of the UGHL develop and implement a policy on this in order to get more HCWs vaccinated and hence reduce the risk of nosocomial hepatitis B infection in the facility. Further studies are recommended on vaccination completion and follow up antibody testing among the HCWs to ascertain the level of protection among vaccinated staff.

### What is known about this topic

- Uptake of hepatitis B vaccine varies in different parts of Africa and Ghana;
- The factors associated with hepatitis B vaccination among healthcare workers differ depending on the setting.

### What this study adds

- The study is the first to determine the hepatitis B vaccine uptake among healthcare workers (HCWs) at the University of Ghana Hospital, Legon (UGHL);
- The study also determines factors associated with hepatitis B vaccination among the HCWs at UGHL, and these include long duration of work and their increased perception of acquiring hepatitis B.

## Competing interests

The authors declare no competing interests.
